# CREB1 activation promotes human papillomavirus oncogene expression and cervical cancer cell transformation

**DOI:** 10.1002/jmv.29025

**Published:** 2023-08-11

**Authors:** Yigen Li, Molly R. Patterson, Ethan L. Morgan, Christopher W. Wasson, Emma L. Ryder, Diego Barba‐Moreno, James A. Scarth, Miao Wang, Andrew Macdonald

**Affiliations:** ^1^ School of Molecular and Cellular Biology, Faculty of Biological Sciences University of Leeds Leeds West Yorkshire UK; ^2^ Astbury Centre for Structural Molecular Biology University of Leeds Leeds West Yorkshire UK; ^3^ School of Life Sciences University of Sussex Falmer Brighton UK; ^4^ Leeds Institute of Rheumatic and Musculoskeletal Medicine, Faculty of Medicine and Health University of Leeds Leeds West Yorkshire UK

**Keywords:** cervical cancer, CREB1, E6, E7, HPV, miR‐203a

## Abstract

Human papillomaviruses (HPVs) infect the oral and anogenital mucosa and can cause cancer. The high‐risk (HR)‐HPV oncoproteins, E6 and E7, hijack cellular factors to promote cell proliferation, delay differentiation and induce genomic instability, thus predisposing infected cells to malignant transformation. cAMP response element (CRE)‐binding protein 1 (CREB1) is a master transcription factor that can function as a proto‐oncogene, the abnormal activity of which is associated with multiple cancers. However, little is known about the interplay between HPV and CREB1 activity in cervical cancer or the productive HPV lifecycle. We show that CREB is activated in productively infected primary keratinocytes and that CREB1 expression and phosphorylation is associated with the progression of HPV+ cervical disease. The depletion of CREB1 or inhibition of CREB1 activity results in decreased cell proliferation and reduced expression of markers of epithelial to mesenchymal transition, coupled with reduced migration in HPV+ cervical cancer cell lines. CREB1 expression is negatively regulated by the tumor suppressor microRNA, miR‐203a, and CREB1 phosphorylation is controlled through the MAPK/MSK pathway. Crucially, CREB1 directly binds the viral promoter to upregulate transcription of the E6/E7 oncogenes, establishing a positive feedback loop between the HPV oncoproteins and CREB1. Our findings demonstrate the oncogenic function of CREB1 in HPV+ cervical cancer and its relationship with the HPV oncogenes.

## 1 INTRODUCTION

Human papillomaviruses (HPV) infection can result in cervical intraepithelial neoplasia (CIN), which left untreated may ultimately progress to cervical cancer, a leading cause of death among women.^1^, ^2^ HPV DNA is detected in more than 95% of cervical cancers, and the high‐risk (HR)‐HPVs, particularly HPV16 and HPV18, are responsible for >70% of invasive cervical cancers.^3^ In addition, HR‐HPVs are also associated with head and neck,^4^ vulvar,^5^ anal,^6^ and penile cancers.^7^

E6 and E7 are the major HR‐HPV encoded oncoproteins and they interact with multiple host factors to deregulate cellular signaling pathways, including p53,^8^, ^9^ pRb/E2F,^10^, ^11^ MAPKs,^12^, ^13^, ^14^ PI3K/Akt,^15^ TGF‐β/Smad,^16^ JAK‐STAT,^17^, ^18^, ^19^ and many others.^20^, ^21^ In addition, the actions of E6 and E7 independently or synergistically contribute to blocking apoptosis in response to DNA damage and other cellular stresses, leading to genomic instability and malignant transformation (reviewed in Scarth et al.^20^).

The transcription factor cAMP response element (CRE)‐binding protein 1 (CREB1) belongs to a subcategory of the basic leucine zipper (bZIP) superfamily and has the potential to regulate approximately 4000 genes.^22^ It can form homo‐ and/or hetero‐dimers with other CREB family members (e.g., ATF1), or the Activator protein 1 (AP‐1) component^23^ to mediate gene transcription by binding to cis‐regulatory elements containing a conserved CRE.^24^ Besides binding to full CRE sequences (TGACGTCA), CREB1 can also bind to half CRE sequences (TGACG or CGTCA) to mediate transcription. The transcriptional activity is induced upon the phosphorylation of CREB1 (S133) by multiple protein kinases including MAPKs,^25^ PKA,^26^ MSKs,^27^, ^28^ and CaMKs.^29^ CREB1 has been shown to function as a proto‐oncogene and its overexpression contributes to human malignancies.^30^, ^31^, ^32^, ^33^ Furthermore, CREB signaling is required for transformation caused by oncogenic viruses, such as human T‐cell leukaemia virus type 1 (HTLV‐1).^34^ However, few studies have focused on CREB1 in cervical cancer and especially in the context of HPV infection. MicroRNAs (miRNAs) are non‐coding RNAs (ncRNAs) that negatively regulate gene expression by binding to the 3′‐untranslated region (UTR) of target messenger ribonucleic acids (mRNAs), leading to degradation of mRNA or translational repression.^35^ Studies have demonstrated that the aberrant expression of miRNAs contribute to cancers.^36^ miR‐203 functions as a tumor suppressor, the ectopic expression of which inhibits carcinogenesis and tumor progression, whereas downregulation of miR‐203 was observed in variety types of cancer due to epigenetic silencing.^37^, ^38^, ^39^ miR‐203 has been shown to be downregulated in cervical cancers and correlated with HPV infection and tumor aggressiveness.^40^, ^41^, ^42^

In this study, we showed CREB1 was overexpressed in HPV+ cervical cancers and promoted cell proliferation and migration. We found that E6‐induced CREB1 phosphorylation and transcriptional activity depended on the MAPK/MSK signaling axis. Additionally, the increased CREB1 expression observed in HPV+ cervical cells was negatively regulated by miR‐203a. Finally, we identified a positive feedback loop between the HPV oncogenes and CREB1, in which CREB1 can directly bind to the viral promoter and upregulate the transcription of HPV oncogenes.

## 2 MATERIALS AND METHODS

### 2.1 Cervical cytology samples

Cervical cytology samples were previously described.^19^ RNA and protein were extracted from the samples using Trizol, following manufacturer's instruction and analysed as described.^12^

### 2.2 HPV positive biopsy samples

Archival paraffin‐embedded cervical biopsy samples were obtained with informed consent. Subsequent analysis of these samples was performed in accordance with approved guidelines, which were approved by Glasgow Royal Infirmary: RN04PC003. HPV presence was confirmed by PCR using GP5+/GP6+ primers.

### 2.3 Cell culture

Cervical cancer cell lines, C33A (HPV−), CaSKi (HPV16+), SiHa (HPV16+), HeLa (HPV18+), SW756 (HPV18+), and HEK293T cells were purchased from American Type Culture Collection and cultured in Dulbecco's modified Eagle's media, supplemented with 10% foetal bovine serum (Thermo Fischer Scientific) and 50U/mL penicillin/streptomycin (Lonza) in a humidified 37°C incubator with 5% CO_2_. Normal human keratinocytes (NHK) and HPV18‐containing NHK were previously described.^43^ Cells were routinely tested for mycoplasma.

### 2.4 Organotypic raft cultures

Control and HPV18 containing foreskin keratinocytes were grown in organotypic raft cultures by seeding the keratinocytes onto collagen beds containing J2‐3T3 fibroblasts. Once confluent the collagen beds were transferred onto metal grids and fed from below with FCS‐containing E media lacking EGF. The cells were allowed to stratify for 14 days before fixing with 4% formaldehyde in E media. The rafts were paraffin‐embedded and 4 μm tissue sections prepared (Propath UK, Ltd.).

### 2.5 Immunohistochemistry

Paraffin embedded organotypic raft culture Section (5 μm) and clinical samples of HPV16 positive CIN lesions (kindly provided by S. Graham, University of Glasgow) were rehydrated and antigens were retrieved by 10 min boiling in sodium citrate buffer (10 mM Tri‐sodium citrate, 0.05% Tween 20, pH 6). Sections were blocked (10% NGS, PBS) for 1 h and incubated with primary and secondary antibodies (1.5% NGS, PBS, 1 h RT). Slides were mounted with DAPI containing agent (Invitrogen) and sealed. Primary antibody P‐CREB (9198, CST) and and secondary antibodies conjugated with Alexa 488 (Invitrogen) were used. The nuclei were counterstained with the DNA stain 4′,6‐diamidino‐2‐phenylindole (DAPI) and mounted in Prolong Gold (Invitrogen).

### 2.6 High calcium differentiation assay

NHK and HPV18 containing keratinocytes were grown in complete E media until 90% confluent. Media was changed to serum free keratinocyte media without supplements (SFM medium, Invitrogen) containing 1.8 mM calcium chloride. Cells were maintained in this media for 72 h before lysis and analysis.

### 2.7 Plasmids, **small interfering RNA** (siRNA), and reagents

Plasmids for HPV oncoproteins have been previously described.^12^ An HPV18 upstream regulatory region (URR)‐driven luciferase reporter construct has been previously described^44^ and was kindly provided by Prof. Felix Hoppe‐Seyler (German Cancer Research Center, Heidelberg, Germany). The HPV16 URR‐driven luciferase reporter construct has been previously described^45^ and was kindly provided by Prof. Iain M. Morgan (Virginia Commonwealth University, Virginia, USA). Pool of four siRNAs targeting CREB1 (No. 1027416) were obtained from QIAGEN. The siRNAs targeting E6 and E7 of HPV16 and HPV18 E6 were previously described.^46^ hsa‐miR‐203a miRNA mimic (MIMAT0000264) was obtained from ABM. Codon optimized HPV18 E6 and E7 sequences were cloned into pcDNA3.1 using KpnI and EcoRI. The 3′‐UTR of CREB1 was cloned into psiCHECK2 using XhoI and NotI. CREB1 was cloned into CMV500 using BamHI and NotI, and pcDNA3.1 using HindIII and EcoRI, respectively. FLAG‐tagged MSK AA has been previously described.^47^ Mutagenesis was performed by PCR using the Site‐directed mutagenesis kit (NEB). The small molecule inhibitors UO126 (MEK1/2 inhibitor, 20 μM), VX‐745 (p38 inhibitor, 10 nM) and SB747651A (MSK inhibitor, 5 μM), and forskolin (30 μM) were purchased from Calbiochem. Lipofectamine™ 2000 (11668019, Invitrogen) or X‐tremeGENE™ (6366236001, Roche) were used for transfection by following the manufacturer's protocol.

### 2.8 Cell proliferation assay

Cell growth curves were performed to evaluate cell proliferation. Briefly, 48 h post‐transfection, cells were trypsinised and re‐seeded. Cells were counted manually using a haemocytometer every 24 h for a period of 5 days.

### 2.9 Colony formation assay

Transfected and corresponding control cells were re‐seeded in a six‐well plate and incubated for 2–3 weeks. Colonies were then stained (1% crystal violet, 25% methanol) and counted manually.

### 2.10 Wound‐healing assay

Wound‐healing assays were performed to evaluate cell migration. Briefly, a scratch was created through the confluent cell monolayer using a plastic micropipette tip. The cells were cultured in low serum (1%) medium and incubated at 37°C for 24 h. Images of wounds were captured using an EVOS microscope. The closure rate was quantified using ImageJ.

### 2.11 Western blot analysis

Equal amounts of protein were separated by sodium dodecyl sulfate–polyacrylamide gel electrophoresis and transferred onto Hybond nitrocellulose membrane (Amersham biosciences), followed by immunoblot analysis. Antibodies used in this study were as follows: CREB1 (9197, CST), phospho‐CREB1 (9198, CST), HPV16 E6 (GTX132686, GeneTex), HPV16 E7 (sc‐65711, Santa Cruz), HPV18 E6 (sc‐365089, Santa Cruz), HPV18 E7 (ab100953, Abcam), Snail (3879, CST), Slug (9585, CST), GAPDH (G‐9, SCBT), GFP (sc‐9996, SCBT), and FLAG (F3165, Sigma). Western blots were visualized with species‐specific HRP conjugated secondary antibodies (Jackson ImmunoResearch) and ECL (Thermo/Pierce).

### 2.12 RNA extraction and qRT‐PCR

Total RNA was extracted using the E.Z.N.A. Total RNA Kit I (Omega Bio‐Tek) or TRIzol reagent (Sigma) according to the manufacturers’ instruction, followed by DNase I treatment (AMPD1, Sigma‐Aldrich). qRT‐PCR was performed using a GoTaq 1‐step qRT‐PCR system (Promega). The reaction was conducted on a CFX96 Connect Real‐Time PCR Detection System (Bio‐Rad) using default protocol with melt curve. B2M and GAPDH served as normalizer genes. miR‐203a expression was detected by miScript PCR system (Qiagen) and Snord68 was used for normalization. The data obtained was analysed using the ΔΔCt method.^48^ Specific primers were used for each gene analysed and are shown in Table S1.

### 2.13 Dual‐luciferase reporter assays

Unless otherwise indicated, the dual‐luciferase reporter assays were conducted in HEK293T. Luciferase activity was examined by using the dual luciferase reporter assay kit (Promega). Briefly, 48 h after transfection, the relative luciferase activity was measured and calculated, according to the reporter system used and as described.^49^

### 2.14 Chromatin immunoprecipitation (ChIP) assays

Cells were fixed with 1% formaldehyde solution for 15 min at RT and fixation quenched by incubation with 125 nM glycine for 5 min. DNA fragments ranging from 200 to 300 bp were generated using sonication. Samples were subjected to immunoprecipitation using anti‐CREB1 or rabbit IgG (ab172730, Abcam) and analysed by qPCR. The PCR primers were as follows: CBS#1 forward 5′‐GTTGTGTTTGTATGTCCTGTGTTTGTG‐3′, reverse 5′‐CCACATAACACACAGAACCACAAAACA‐3′; AP‐1E forward 5′‐CGGTTGCCTTTGGCTTATGTC‐3′, reverse 5′‐GTTATGCAAGCAATTGTTGTAGCGCA‐3′; AP‐1P forward 5′‐GCTAATTGCATACTTGGCTTGTACAAC‐3′, reverse 5′‐GTGCTGCCCAACCTATTTCGG‐3′; CBS#2 forward 5′‐GTAACCGAAAACGGTCGGG‐3′, reverse 5′‐CAGGTAGCTTGTAGGGTCGC‐3′. Fold enrichment was calculated by comparing to the IgG isotype control.

### 2.15 Microarray analysis

For microarray analysis of CREB1 expression, the following datasets were used: GSE6791, GSE63514, and GSE39001. For microarray analysis of miR‐203a expression, the following datasets were used: GSE30656 and GSE19611.

### 2.16 Statistical analysis

Statistical analyses were performed using GraphPad Prism 7.00. The Student *t*‐test (unpaired, two‐tailed) was performed to determine significance.

## 3 RESULTS

### 3.1 CREB1 is activated in HPV containing keratinocytes and cervical cancer progression

**Figure 1:**
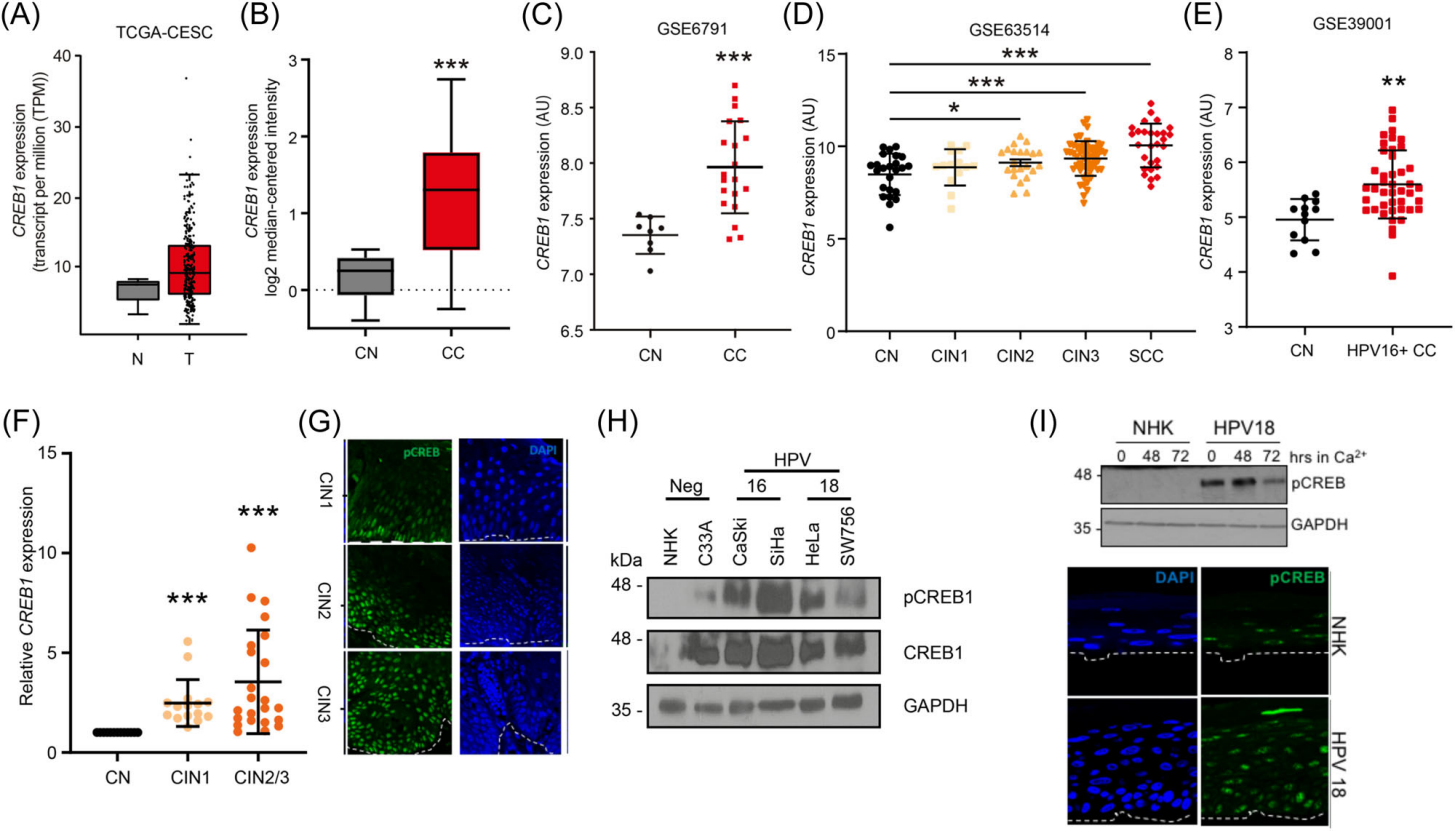
CREB1 is overexpressed in HPV containing primary keratinocytes, HPV+ cervical cancers and is associated with disease progression. (A) TCGA data analysis of *CREB1* expression in cervical squamous cell carcinoma and endocervical adenocarcinoma (CESC); (B) Pyeon multicancer mRNA dataset analysis of *CREB1* in cervical cancer (CC) was compared to cervical normal (CN); (C) GSE6791 dataset analysis of *CREB1* expression in CC tissue was compared to CN tissue; (D) GSE63514 dataset analysis of *CREB1* expression was compared among different cervical intraepithelial neoplasia (CIN) grades and cervical squamous cell carcinoma (SCC). CIN grades represent the severity of cervical disease; (E) GSE39001 dataset analysis of *CREB1* expression in HPV16+ CC tissue was compared to CN tissue; (F) qPCR analysis of *CREB1* in cervical cytology samples collected from healthy patients and patients with different CIN grades; (G) Representative immunostaining analysis of tissue sections from cervical lesions representing low‐grade through to high‐grade cervical disease. Sections were stained for phosphorylated CREB1 (green) and nuclei were visualized using DAPI (blue). Images were acquired using identical exposure time. (H) Western blot analysis of CREB1 in HPV+ cervical cancer cell lines compared to NHK; (I) Representative western blot analysis of normal human keratinocytes (NHK) and HPV18‐containing keratinocytes subjected to high calcium differentiation and analysed for CREB phosphorylation. GAPDH serves as loading control. Representative sections of organotypic raft cultures from NHK and HPV18‐containing keratinocytes stained with antibodies specific for phosphorylated CREB (green) and counterstained with DAPI to highlight the nuclei (blue). Images were acquired using identical exposure times. White dotted lines indicate the basal cell layer. Data shown are mean ± *SD*, *n* > 3. *p < 0.05; **p < 0.01; ***p < 0.001. CIN, cervical intraepithelial neoplasia; HPV, human papillomaviruses; mRNA, messenger ribonucleic acid.

To understand if CREB1 plays a role in the biology of cervical cancer, we explored the TCGA database by Gene Expression Profiling Interactive Analysis (GEPIA) and found that *CREB1* expression in cervical cancers was higher than normal tissue (Figure 1A). Consistently, according to the OncoMine database and public dataset (GSE6791), we found significantly upregulated *CREB1* expression in cervical cancers compared with normal cervical tissue (Figure 1B,C). We also explored GSE datasets for *CREB1* expression in different CIN grades, representing disease severity, and found that *CREB1* expression significantly correlated with increasing CIN grade and was further increased in cervical squamous cell carcinoma (Figure 1D). In addition, we investigated *CREB1* expression with regards to HPV infection status. By analysis of public dataset (GSE39001), we found a significant higher *CREB1* expression in HPV16+ cervical cancer specimens compared with healthy exocervix (Figure 1E).

To experimentally confirm these findings, we harvested cervical cytology samples collected from healthy patients as negative controls and patients with increasing CIN grade. Our results showed that *CREB1* expression was significantly upregulated with CIN progression (Figure 1F). These findings were corroborated using immunostaining for the active, phosphorylated, form of CREB in sections of tissue from low‐grade CIN1 and high‐grade CIN3 samples, revealing a marked increase in phosphorylated CREB in CIN3 (Figure 1G). Western blot analysis of a panel of cervical cancer cell lines demonstrated that CREB1 expression was upregulated in HPV16+ (CaSKi and SiHa) and HPV18+ (HeLa and SW756) transformed cervical cancer cell lines compared with primary NHK (Figure 1G). CREB1 phosphorylation was also increased in these HPV+ cell lines, suggestive of increased transactivation activity (Figure 1H). Levels of CREB phosphorylation were also measured in NHK and HPV18‐containing cells by western blotting. Uninfected cells had very low levels of basal CREB phosphorylation compared to keratinocytes harboring HPV18 (Figure 1I—compare lanes 1 and 4). To ascertain whether CREB phosphorylation was further altered during keratinocyte differentiation, monolayer cultures were cultured in high calcium media for 72 h and samples taken for western blot analysis. Whilst subject to a decline, enhanced CREB phosphorylation was maintained at detectable levels in the HPV18‐containing keratinocytes during differentiation (Figure 1I—compare lanes 2 and 5, 3 and 6). Next we confirmed our findings in a second model of keratinocyte differentiation. NHK and HPV18‐containing keratinocytes were stratified in organotypic raft culture for 14 days, this method recapitulates all stages of the HPV lifecycle.^17^, ^43^ Raft sections were stained with an antibody detecting phosphorylated CREB (representative sections shown in Figure 1I). Staining for phosphorylated CREB was evident in both the basal and suprabasal layers of the NHK and HPV18‐containing cells. However, the levels of CREB phosphorylation were elevated in the presence of HPV18. Taken together, our results showed that CREB1 expression increased with the progression of cervical disease severity and that CREB1 protein and phosphorylation levels were also increased in HPV+ cell lines and in primary cells containing HPV18.

### 3.2 CREB1 drives proliferation and clonogenicity but not apoptosis of HPV+ cervical cancer cells

**Figure 2:**
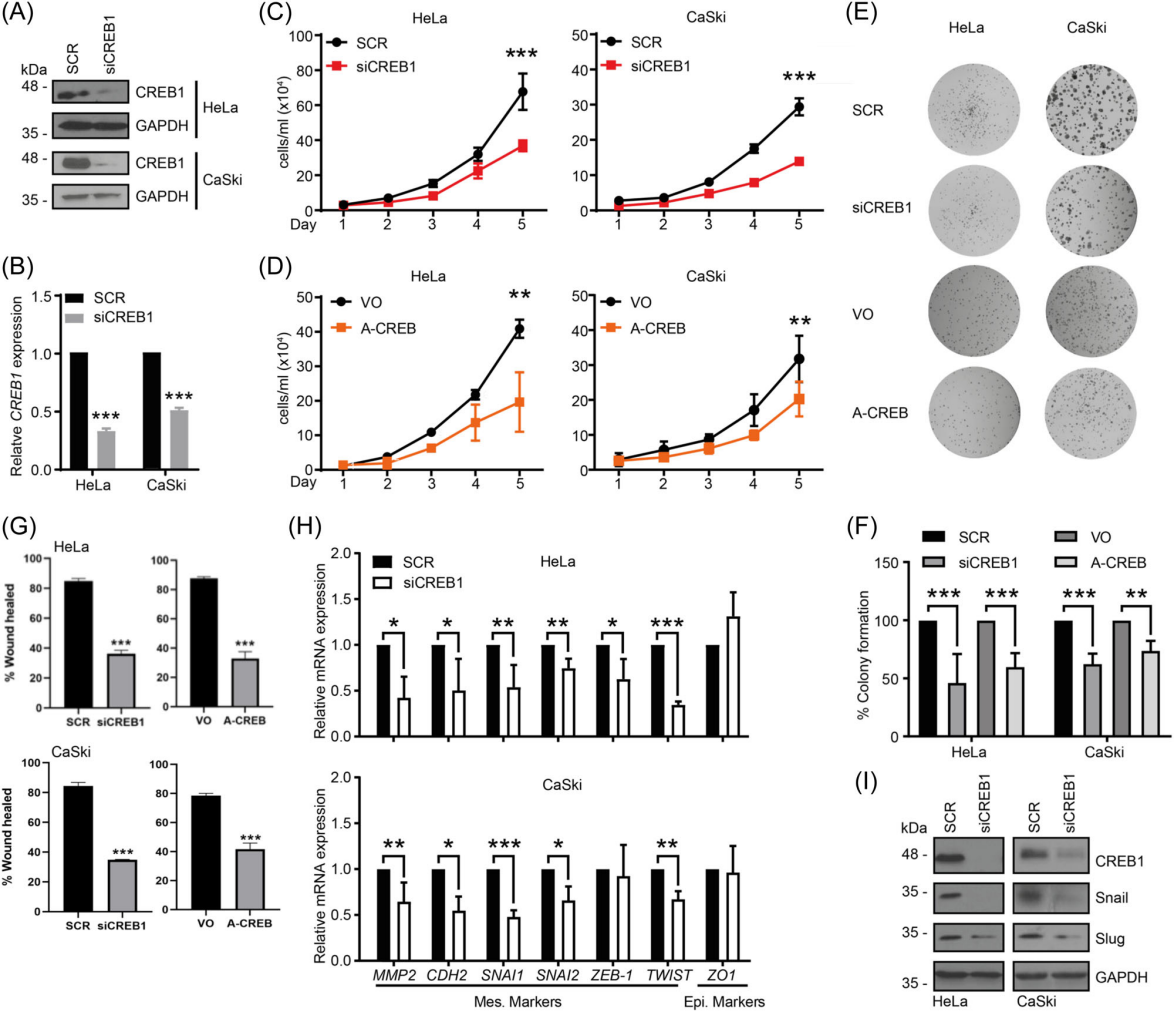
Depletion of CREB1 inhibits the proliferation, migration and EMT of cervical cancer cells in vitro. (A) Western blot analysis of CREB1 in indicated cell lines transfected with a pool of four siRNAs targeting CREB1 (siCREB1). GAPDH loading control; (B) qPCR analysis of *CREB1* in indicated cell lines transfected with siCREB1; (C, D) Cell proliferation analysed by cell growth curve in indicated cell lines transfected with siCREB1, or A‐CREB, a dominant negative inhibitor for CREB1; (E,F) Representative images for colony formation assay performed in indicated cell lines transfected with siCREB1 or A‐CREB. Quantification of (E) as shown in (F); (G) Quantification of wound closure as an evaluation for cell migration in indicated cell lines transfected with siCREB1 or A‐CREB; (H) qPCR analysis of EMT markers in indicated cell lines transfected with siCREB1; (I) Western blot analysis of EMT markers in indicated cell lines transfected with siCREB1. Data shown are mean ± *SD*, *n* ≥ 3. *p < 0.05; **p < 0.01; ***p < 0.001. EMT, epithelial to mesenchymal transition; GAPDH, glyceraldehyde 3‐phosphate dehydrogenase; siRNA, small interfering RNA.

To understand whether CREB1 plays a role in cervical cancer cell biology, we performed in vitro experiments to evaluate cell proliferation and clonogenicity in HPV+ cell lines. Cells were transfected with a pool of four siRNAs targeting CREB1 and knockdown efficiency was verified before seeding for all proliferation assays (Figure 2A,B), with results showing that silencing of CREB1 in HeLa and CaSKi significantly inhibited cell growth (Figure 2C). In addition to depleting CREB1 from cells, we also utilized overexpression of A‐CREB, an engineered dominant negative inhibitor of CREB1 activity^50^ (Figure S1A). The mRNA levels of *FOS*, *NR4A1* and *NR4A3*, which are *bone fide* CREB1‐dependent genes containing CRE sequences within their promoter regions,^28^, ^51^ were reduced upon A‐CREB overexpression, confirming the successful inhibition of CREB1 transcriptional activity (Figure S1B). In agreement with the CREB1 siRNA data, cell proliferation was also inhibited by A‐CREB overexpression (Figure 2D). Consistently, fewer colonies were formed in cells treated with siCREB1 or overexpressing A‐CREB than those in the controls (Figure 2E,F). To rule out that the observed changes in cell number and clonogenicity were not a result of increased apoptosis due to the inhibition or loss of CREB we performed western blot analysis in cells transfected with siRNA targeting CREB or A‐CREB for the proteolytic cleavage of PARP (Figure S1C,D). We observed no increase in the presence of a faster migrating PARP band, indicative of apoptosis. To be certain we performed Annexin V assays, which also revealed no difference in apoptosis levels between control and CREB depleted cells (Figure S1E). These results suggested that inhibition of CREB1 suppressed cell proliferation but did not increase apoptosis in HPV+ cervical cancer cells.

### 3.3 Inhibition of CREB1 suppresses cell migration and epithelial mesenchymal transition (EMT)

EMT plays an important role in cervical cancer progression and metastasis^52^ and CREB1 has been reported to promote migration and EMT in cancers.^53^, ^54^ To address this, we first performed wound healing assays to investigate the effect of CREB1 on the migration of cervical cancer cells. CREB1 silencing or inhibition significantly slowed the wound closure rate compared with the control (Figure 2G and Figure S1F). These data indicated that inhibition of CREB1 attenuated migration in cervical cancer cells.

To determine the role of CREB1 in regulating EMT, we first investigated the changes in expression of multiple EMT markers by RT‐qPCR after CREB1 silencing. This showed that depletion of CREB1 resulted in reduced mRNA levels of mesenchymal markers including *MMP2*, *CDH2* (N‐cadherin), *SNAI1* (Snail), *SNAI2* (Slug), and *TWIST1* in both HeLa and CaSKi cells (Figure 2H). To validate these findings, we analysed the protein expression of Slug and Snail, key transcription factors which regulate EMT.^55^, ^56^ Consistently, we showed that their protein expression was decreased with CREB1 silencing (Figure 2I). These results indicated that CREB1 contributed to migration and EMT in cervical cancer cells.

### 3.4 CREB1 contributes to HPV18 E6 driven proliferation

**Figure 3:**
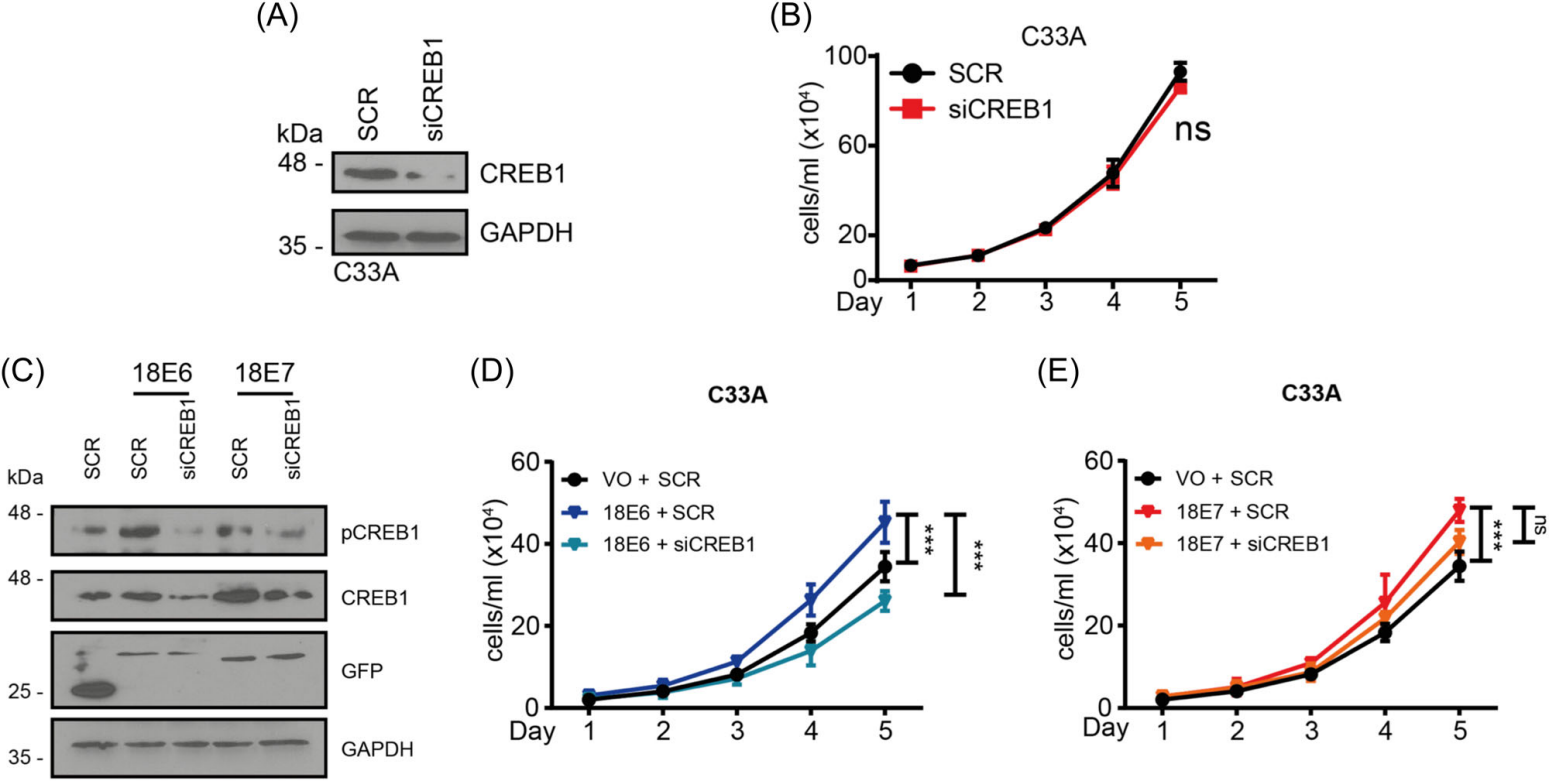
HPV18 E6 promotes proliferation in HPV‐cervical cancer cell line C33A *via* CREB1. (A) Western blot analysis of CREB1 in HPV‐CC cell line, C33A, transfected with siCREB1; (B) Cell proliferation analysed by cell growth curve in HPV‐CC cell line, C33A, transfected with siCREB1; (C) Western blot analysis in C33A, co‐transfected with siCREB1 and GFP‐HPV18 E6 or E7; (D, E) Cell proliferation analysed by cell growth curve in C33A, co‐transfected with siCREB1 and HPV18 E6 or E7. Data shown are mean ± *SD*, *n* = 3. ns, not significant; ***p < 0.001. CC, cervical cancer; HPV, human papillomaviruses.

Expression of the HPV oncoproteins is necessary for cervical cancer cell proliferation in cell culture. To determine whether CREB1 was required for proliferation by HPV18 oncoproteins, C33A cells were transfected with GFP‐tagged HPV18 E6 and E7. Of note, although C33A cells showed increased CREB1 expression and phosphorylation compared with NHK controls (Figure 1H), silencing CREB1 did not inhibit the proliferation (Figure 3A,B). Expression of 18E6 led to an increase in both CREB1 protein levels and phosphorylation over the baseline levels seen in C33A cells, whereas 18E7 expression only resulted in an increase in CREB1 protein levels (Figure 3C). Western blot demonstrated that CREB1 siRNAs‐mediated knockdown was successful in both of these cell lines (Figure 3C). C33A cells expressing 18E6 and 18E7 showed increased proliferative capacity over empty vector controls and their growth was reproducibly impaired when CREB1 was silenced, with the loss of CREB1 having a more pronounced impact on 18E6 driven proliferation compared to 18E7 mediated growth (Figure 3D,E). The results suggested that whilst CREB1 expression was dispensable for proliferation in HPV‐C33A cell it was required for growth driven by the HPV18 oncoproteins, particularly E6.

### 3.5 HPV E6 induces CREB1 phosphorylation and activity by a MAPK/MSK signaling pathway

**Figure 4:**
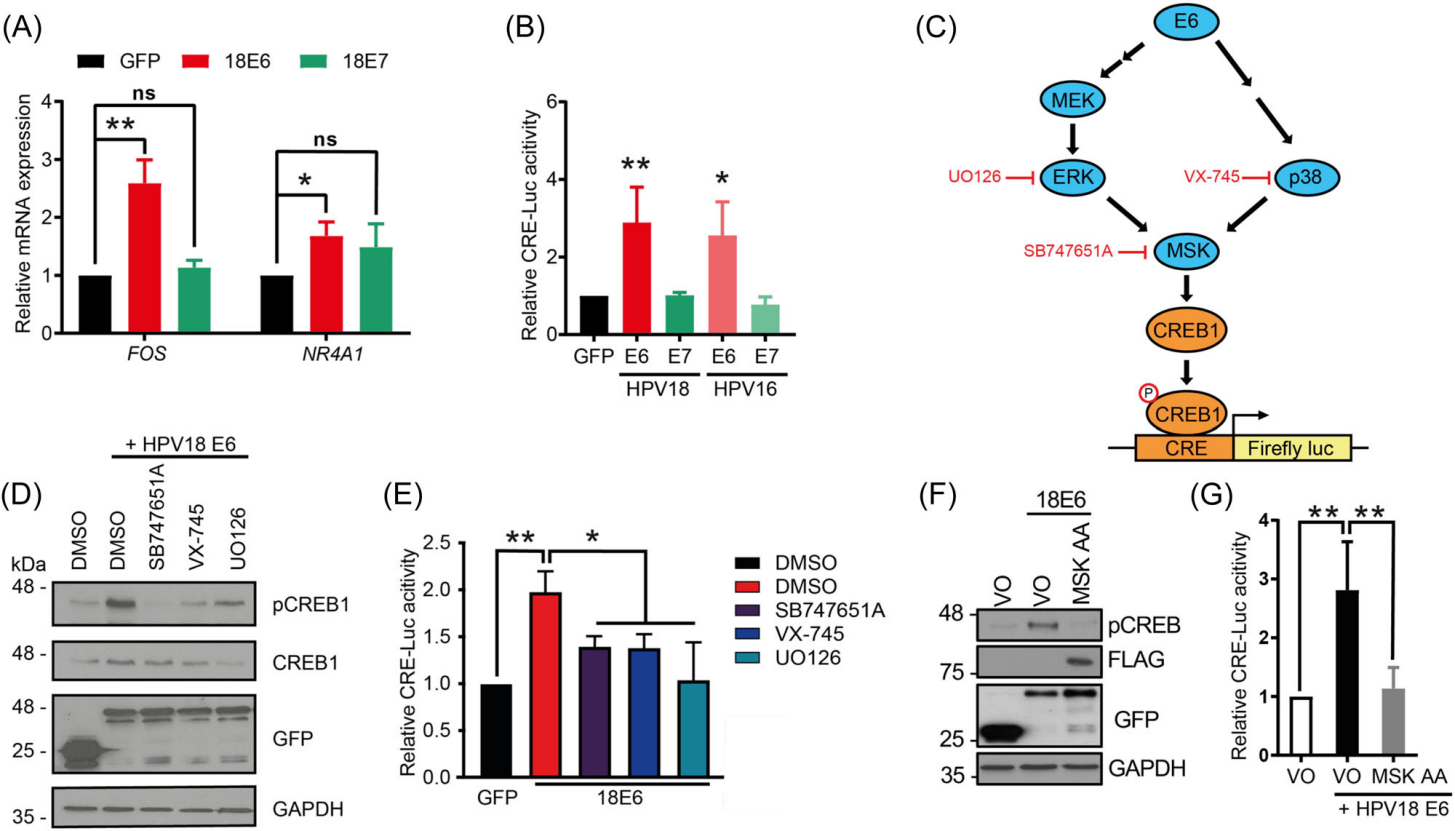
HPV E6 induces CREB1 activity *via* MAPK/MSK signaling. (A) qPCR analysis of CREB1‐dependent genes in C33A transfected with HPV18 E6 or E7; (B) Dual luciferase CRE‐driven luciferase reporter analysis in HEK293T transfected with HPV E6 or E7; (C) Schematic of inhibitors used for E6‐activating MAPK signaling pathways; (D, E) Western blot analysis and dual luciferase CRE‐driven luciferase reporter analysis in HEK293T transfected with HPV18 E6 and SB747651A (5 µM), VX‐745 (10 nM), or UO126 (20 µM); (F, G) Western blot analysis and dual luciferase CRE‐driven luciferase reporter analysis in C33A and HEK293T co‐transfected with HPV18 E6 and dominant negative mutant of MSK, T581A/T700A (MSK AA). Data shown are mean ± *SD*, *n* = 3. ns, no significant; *p < 0.05; **p < 0.01. CC, cervical cancer; HPV, human papillomaviruses.

To investigate which HPV oncoprotein was responsible for increasing CREB1 transactivation function, we again utilized C33A cells and evaluated CREB1 activity by detecting the mRNA levels of the CREB1‐dependent genes *FOS* and *NR4A1*, following overexpression of 18E6 or 18E7. This showed significant upregulation of *FOS* and *NR4A1* mRNA in cells expressing 18E6 but not 18E7 (Figure 4A and Figure S2A). In tandem, we employed a CRE‐driven luciferase reporter system, the results of which demonstrated significantly enhanced luciferase levels in cells expressing E6, but not E7 (Figure 4B and Figure S2B). These results identified HPV E6 as the primary mediator of CREB1 activation. In a complimentary analysis we demonstrated that our panel of CREB1‐dependent genes (*FOS, NR4A1, NR4A3*) showed increased expression in primary keratinocytes containing HPV18 (Figure S2C). Thus, cells expressing HPV oncoproteins in isolation or the whole genome demonstrate evidence of increased CREB1 transcriptional activation.

HPV E6 has been demonstrated to deregulate MAPK signaling.^12^, ^13^, ^14^ To investigate whether E6‐induced CREB1 activity was ERK/p38 kinase dependent, we performed the CRE‐driven luciferase assay in 18E6 expressing cells treated with small molecule inhibitors targeting the MAPK signaling components (Figure 4C). The results showed that inhibition of either ERK, p38, or the downstream effector MSK^57^ reduced E6‐induced CREB phosphorylation and CRE‐driven luciferase activity (Figure 4D,E). To orthogonally confirm the importance of MSK in E6‐driven CREB1 phosphorylation and activation, we used a characterized MSK mutant (MSK T581A/T700A (AA)), which abolishes MSK activity,^47^ and found that in agreement with our pharmacological data, the increase in CREB1 phosphorylation and CRE‐driven luciferase levels mediated by 18E6 was impaired by the MSK mutant (Figure 4F,G). Taken together, these results suggested that HPV E6 induced CREB1 activity via MAPK/MSK signaling.

### 3.6 miR‐203a directly targets and inhibits CREB1 expression

**Figure 5:**
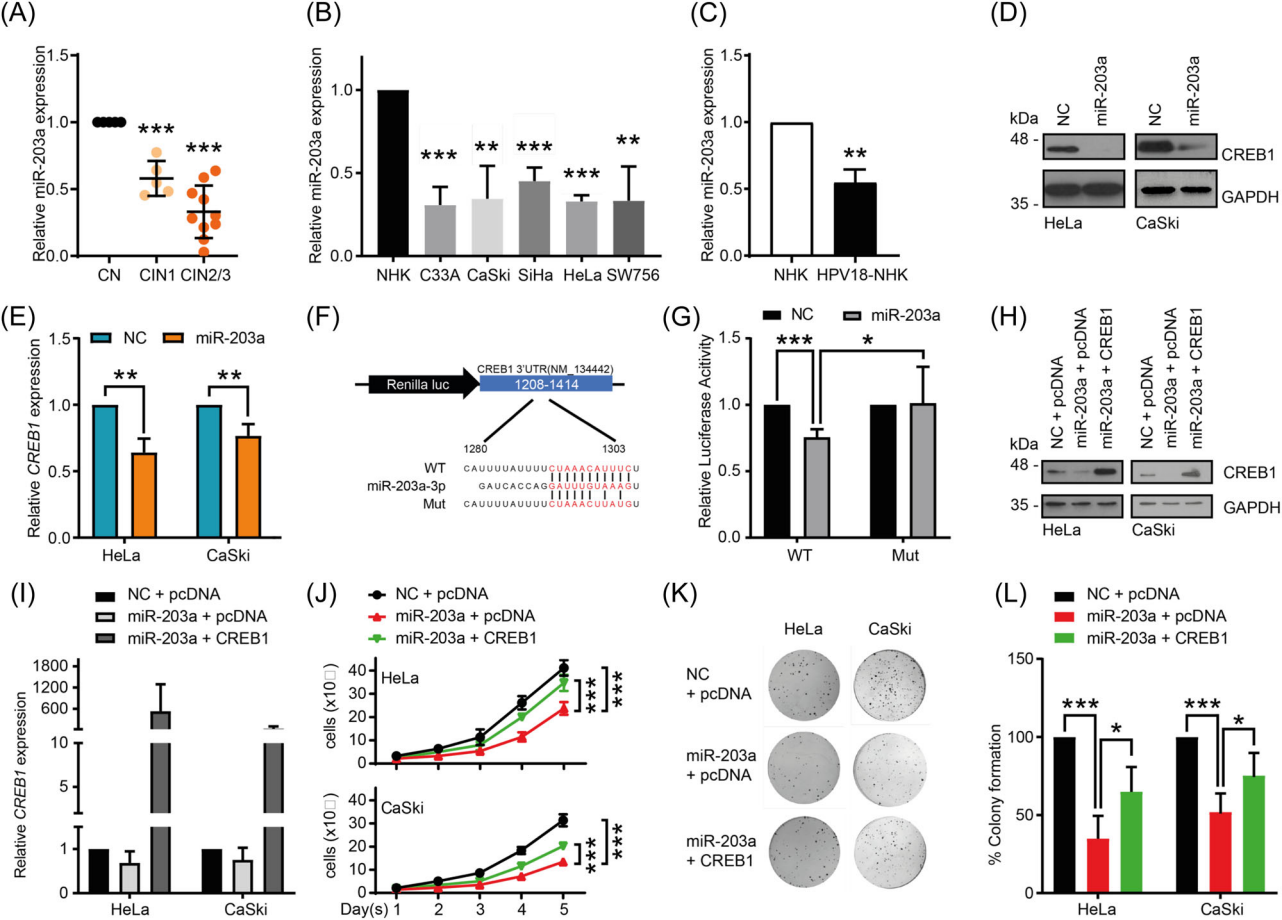
miR‐203a overexpression attenuates cell proliferation by directly targeting CREB1. (A) qPCR analysis of miR‐203a in cervical cytology samples from patients with different CIN grades; (B) qPCR analysis of miR‐203a expression in CC cell lines compared to NHK; (C) qPCR analysis of miR‐203a in HPV18‐transformed NHK; (D, E) Western blot and qPCR analysis in indicated cells transfected with miR‐203a mimic; (F) Schematic of predicted miR‐203a binding sites within CREB1 3′‐UTR and point mutation introduced, fused to luciferase reporter system; (G) Dual luciferase reporter analysis of CREB1 3′‐UTR (WT and Mut) ‐controlled activity in HEK293T with overexpressing miR‐203a mimic; (H, I) Western blot and qPCR analysis of CREB1 in indicated cells co‐transfected with miR‐203a mimic and CREB1; (J–L) Cell proliferation and clonogenicity assessed by cell growth curve and colony formation assay in indicated cells co‐transfected with miR‐203a mimics and CREB1. Data shown are mean ± *SD*, *n* = 3. *p < 0.05; **p < 0.01; ***p < 0.001. CC, cervical cancer; HPV, human papillomaviruses; UTR, untranslated region.

During our studies we noted that CREB1 protein levels were also increased in HPV+ cells (Figure 1F and 1H). Whilst the importance of CREB1 phosphorylation and the means by which this is achieved are better characterized, the control of CREB1 protein expression is less well understood. This prompted us to investigate the underlying mechanisms driving the increased CREB1 expression. Amongst their many modes of gene regulation, the HPV oncoproteins can modulate the miRNA network to manipulate the post‐transcriptional regulation of target genes both in virus infection and in cancer.^58^ Using TargetScan,^59^ we noted a highly conserved miR‐203a binding site within the *CREB1* 3′‐UTR. Further analysis of cervical cytology samples and the GSE30656 and GSE19611 datasets showed that miR‐203a was downregulated with CIN progression (Figure 5A) and in cervical cancer (Figure S3A,B), which correlates with miR‐203a serving as a tumor suppressor. In agreement with previous studies showing that miR‐203a expression is modulated by HPV,^42^ our analysis showed that the expression of miR‐203a in HPV+ cervical cancer cell lines was significantly lower compared with NHK (Figure 5B), and the presence of the whole HPV18 genome in NHK was sufficient to reduce miR‐203a expression (Figure 5C).

To experimentally validate whether miR‐203a inhibits CREB1 expression, a miR‐203a mimic was employed (Figure S3C). Endogenous CREB1 protein and mRNA expression were both significantly reduced with miR‐203a overexpression (Figure 5D,E). We then used a luciferase reporter controlled by a partial *CREB1* 3′‐UTR containing the putative miR‐203a binding site (Figure 5F). Overexpression of the miR‐203a mimic significantly decreased the luciferase activity controlled by the wild‐type (WT) *CREB1* 3′‐UTR, whereas it failed to repress the activity of a luciferase reporter containing a mutated miR‐203a binding sequence in the 3′‐UTR (Mut) (Figure 5G). Taken together, the above results confirmed that miR‐203a directly targeted the *CREB1* 3′UTR and repressed CREB1 expression.

### 3.7 miR‐203a overexpression attenuates cell proliferation by targeting CREB1

To determine whether the tumor suppressive effects of miR‐203a are due to its impact on CREB1 expression, we co‐expressed a miR‐203a mimic and CREB1 (Figure 5H,I). Our results showed that cervical cancer cells overexpressing the miR‐203a mimic alone presented significantly suppressed cell growth and clonogenicity, whilst overexpression of CREB1 could partially rescue the suppression in HeLa and CaSKi cells (Figure 5J–L). These results revealed that miR‐203a‐attenuated cell proliferation was at least partially due to targeting of CREB1.

### 3.8 CREB1 transcriptionally upregulates HPV oncoprotein expression by binding to the HPV URR

**Figure 6:**
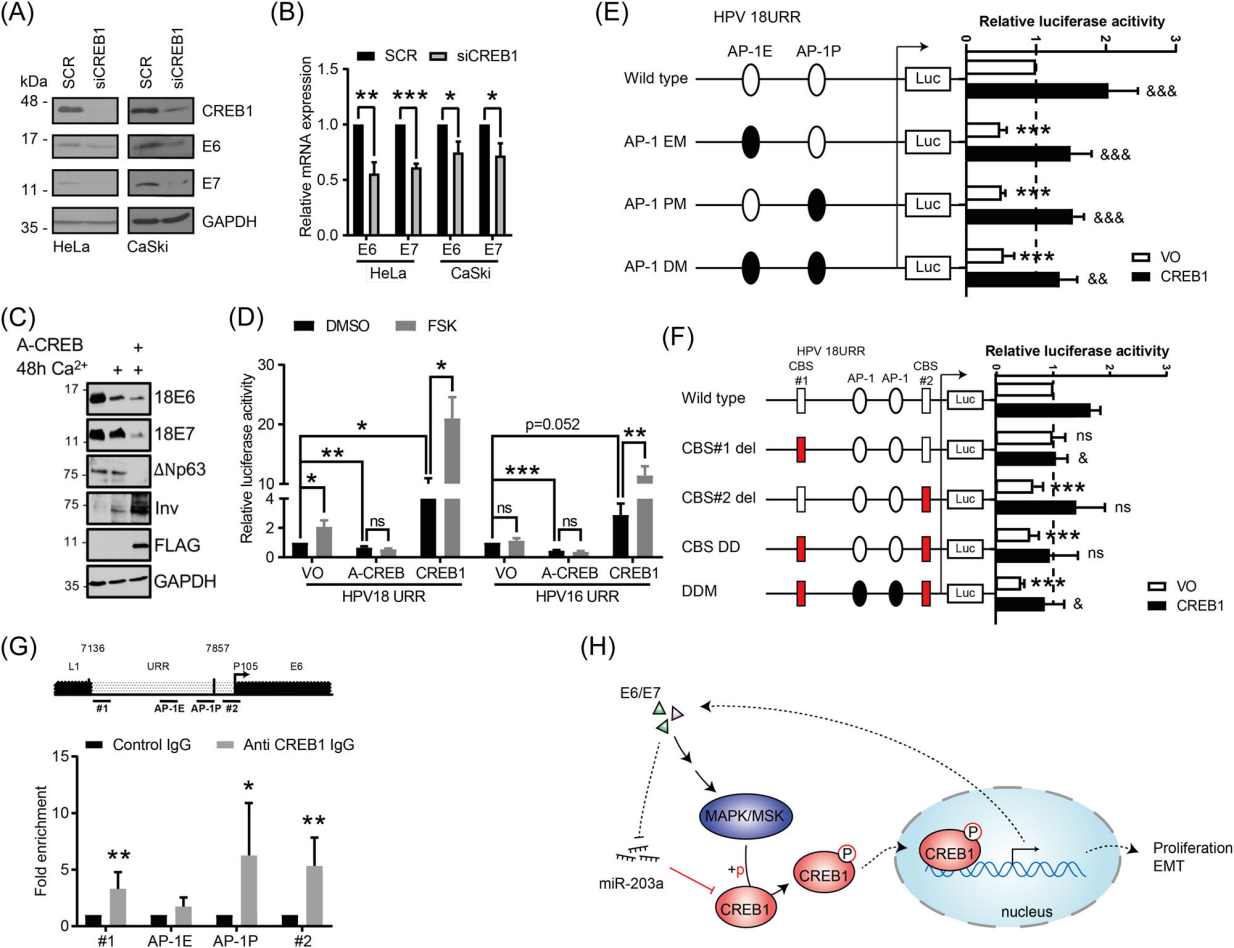
CREB1 transcriptionally upregulates HPV oncoprotein expression by binding to the HPV URR. (A, B) Western blot and qPCR analysis of E6 and E7 expression in indicated cells transfected with siCREB1; (C) Primary HPV18 containing keratinocytes control and A‐CREB transfected and differentiated in high calcium, probed for E6 and E7 oncoproteins, p63 and involucrin as markers of proliferation/differentiation, FLAG to confirm A‐CREB expression and GAPDH as a loading control. (D) Dual luciferase HPV16/18 URR‐driven luciferase reporter analysis in HEK293T transfected with A‐CREB or CREB1, and/or stimulated with Forskolin (FSK), which promotes CREB1 activity; (E) Dual luciferase reporter analysis of HPV18 URR wild type, mutation of AP‐1 site within enhancer region (AP‐1 EM), promoter region (AP1‐PM), or both sites (AP1‐DM) activity in HEK293T with overexpressing CREB1. *, compared with wild type transfected with Vector only (VO); &, compared with the corresponding reporter construct transfected with VO. The basal transcriptional activity was set to 1; (F) Dual luciferase reporter analysis of HPV18 URR wild type, deletion of putative CREB1‐binding site 1(CBS#1 del), CBS#2 del, deletion of both sites (DD), or AP‐1 mutants with CBSs DD (DDM) activity in HEK293T with overexpressing CREB1. *, compared with wild type transfected with VO; &, compared with wild type reporter construct with overexpressing CREB1. The basal transcriptional activity was set to 1; (G) ChIP analysis of direct interaction between CREB1 with HPV18 URR. Four regions were detected: CBS#1 (#1), 7169–7313; AP‐1E, 7608–7614; AP‐1P, 7792–7798; CBS#2 (#2), 38–150; (H) Schematic of proposed model. Data shown are mean ± *SD*, *n* ≥ 3. ns, non‐significance. *p < 0.05; **p < 0.01; ***p < 0.001. HPV, human papillomaviruses; URR, upstream regulatory region.

During our studies we noticed that the depletion of CREB1 in the HPV+ cancer lines decreased E6 and E7 expression (Figure 6A,B). This effect was also seen in the context of a productive infection. When keratinocytes containing HPV18 were transfected with A‐CREB and induced to differentiate with high calcium, E6 and E7 levels were both reduced compared to control. We also observed a concomitant reduction in proliferation markers such as ΔNp63 and a corresponding increase in expression of terminal differentiation markers like involucrin (Figure 6C). Given this broad effect on oncoprotein expression, we wondered whether CREB1 could regulate the transcription of E6 and E7 via the URR. To investigate this, cells were co‐transfected with luciferase reporters driven by the URR of either HPV16 or HPV18, and A‐CREB or CREB1 (Figure S3D), and stimulated by Forskolin (FSK), which promotes CREB1 activity. The results showed that both HPV16 and HPV18 URR activity was significantly enhanced by CREB1 overexpression and FSK, and diminished by A‐CREB even in cells stimulated by FSK (Figure 6D). These results implied that CREB1 contributed to HPV URR activity. Although there is no established consensus CRE sequence reported within HPV16/18 URRs, we and others have previously showed AP‐1 regulated HPV URR activity.^12^, ^44^ Therefore, we wondered whether the AP‐1 sites contribute to CREB1‐mediated URR activity as CREB1 can regulate gene transcription via CRE and AP‐1 binding sites.^60^, ^61^ To investigate this, we co‐transfected cells with CREB1 and a luciferase reporter containing WT or mutants of the reported AP‐1 sites within the HPV18 URR. Our results showed that mutation of either or both AP‐1 sites within the enhancer region (AP‐1E) or promoter region (AP‐1P) suppressed the basal URR activity, while CREB1 overexpression was able to enhance the URR activity, exceeding the basal level detected with the WT reporter but not reaching the levels reached by the WT reporter in cells overexpressing CREB1 (Figure 6E). This implied the upregulation of HPV18 URR activity by CREB1 might be partially dependent on the AP‐1 sites, but that there must be additional regions within the URR that are regulated by CREB1.

By using JASPAR,^62^ two putative CREB1 binding sites (CBSs) #1 (7202–7214) and #2 (76–88) were predicted, as indicated in Figure 6F. To test their functional relevance, we generated deletion mutants of the CBSs along with the AP‐1 mutants within the HPV18 URR luciferase reporter plasmid. The results demonstrated that deletion of CBS#1 alone had no impact on basal URR activity, but prevented any subsequent increase in CREB1‐induced URR activity (Figure 6F). In contrast, deletion of CBS#2 alone, double deletion (DD), or double deletion with AP‐1 site mutations (DDM) significantly suppressed basal URR activity (Figure 6F). This suggested CBS#2, but not CBS#1, contributed to basal expression driven by the URR. Furthermore, the other mutants containing CBS#1 could reduce CREB1‐induced URR activity compared to the wild type. These results implied CBS#1 may have an effect on regulating the URR activity under CREB1 activity and CBS#2 may regulate basal URR activity. To further investigate the regulatory function of CREB1 on the HPV18 URR, we performed a ChIP‐qPCR assay. We observed an enrichment of CREB1 at both of the putative CBSs, as well as at the AP‐1 binding site situated within the URR (Figure 6G), suggesting a direct interaction between CREB1 and the HPV18 URR. Taken together, our results indicated that CREB1 upregulated transcription of the HPV oncogenes by direct binding to the HPV18 URR.

## 4 DISCUSSION

CREB1 is a multifunctional transcription factor with the potential to regulate approximately 4000 target genes.^22^ Overexpression of CREB1 is often found in multiple human cancers and linked to several hallmarks of cancer.^63^ Although CREB1 was previously reported to regulate mitophagy in cervical cancer,^64^ no investigation has been taken to study CREB1 function in the context of HPV infection, and so its functions remain to be fully elucidated in these cells. Here, we showed CREB1 expression positively correlated with cervical disease progression. Using RNAi knockdown and the dominant negative CREB1 inhibitor A‐CREB, we demonstrated that CREB1 functions as a proto‐oncogene by promoting proliferation, migration and EMT in HPV+ cervical cancer cells. We did not observe a role for CREB1 in regulating apoptosis in the cervical cancer cells, which would have provided an alternative explanation for the growth curve and clonogenicity assays undertaken. We did not test for senescence and so cannot rule out that CREB signaling might also feed into this biological process. HPV E6 and E7 have been shown to regulate the MAPK signaling pathway,^12^, ^13^, ^14^ which are direct drivers of CREB1 activation through their ability to phosphorylate and activate the MAPK‐activated kinase MSK,^27^, ^28^ which phosphorylates CREB1 on S133.^25^ We found CREB1 phosphorylation and activity was upregulated by HPV E6, and it required the MAPK/MSK signaling axis. Furthermore, E6‐enhanced cell proliferation in the HPV‐cervical cancer C33A cell line was at least partially CREB1‐dependent. Taken together, our results suggest E6 utilizes the MAPK/MSK pathway to activate CREB1, thereby driving cervical cancer. We also demonstrated that active CREB1 was necessary to maintain proliferative signaling within the differentiating environment of the epithelium, as loss of CREB activity correlated with a reduction in proliferation markers such as p63 and increased expression of terminal differentiation marker expression. This is likely mediated through several of the myriad of gene targets of CREB1, many of which such as cFos are known to induce proliferation and have been associated with HPV previously. Going forward it would be informative to determine the impact of CREB inactivation, either through knockdown or A‐CREB expression, more comprehensively on productive infection.

Non‐coding RNAs (ncRNAs), including miRNAs, regulate chromatin remodeling, transcription, post‐transcriptional modifications, and signal transduction and thereby control many fundamental pathological processes.^65^ Studies have indicated that deregulated expression of ncRNAs is pivotal to HPV+ cervical cancer.^16^, ^46^, ^66^, ^67^ For example, the oncogenic miR18a targets the STK4 tumor suppressor to inhibit the Hippo pathway and activate the protumorigenic transcription factor YAP1.^46^ miR‐203a, a well‐studied tumor suppressor, is downregulated by the HPV oncoproteins^41^, ^42^, ^68^ and controls the pathogenesis of cervical cancer by regulating multiple target genes including *VEGF*,^69^ *BANF1*,^70^ and *ZEB1*.^71^ An inverse correlation between miR‐203a and *CREB1* expression was observed in melanoma.^72^, ^73^ However, to our knowledge, no evidence of their relationship in cervical cancer has been shown. In the present study, we confirmed that CREB1 is a direct miR‐203a target in cervical cancer cells. Our results demonstrated CREB1 overexpression could partially rescue miR‐203a‐suppressed proliferation, suggesting the importance of miR‐203a/CREB1 in regulating cervical cancer.

HPV early gene transcription is initiated from the early promoter located upstream of the E6 open reading frame (P97 for HPV16 and P105 for HPV18) within the viral URR. Multiple host cell transcription factors have been shown to bind the URR to control early gene transcription, such as AP‐1, SP1, TBP, Oct‐1, and YY1.^74^ CREB1 has been reported to regulate transcription of viral genes, including those of HTLV‐1,^75^ Kaposi's sarcoma‐associated herpesvirus,^76^ HBV,^77^ human immunodeficiency virus,^78^ and EBV.^79^ However, to our knowledge, there is no report demonstrating that CREB1 can directly regulate HPV gene transcription. Here, we showed that the transcription of HPV early genes was upregulated by CREB1 in cancer cell lines and in primary keratinocytes harboring the entire HPV18 genome. Mechanistically, we identified two putative CBSs responsible for CREB1 binding and CREB1‐induced URR transcriptional activity. Recently, PKA, a direct upstream activator of CREB1, and Forskolin, a stimulator of the PKA/CREB1 signaling, were shown to regulate the replication of HPV18.^80^ We discovered that overexpression of A‐CREB could reduce the Forskolin‐mediated enhancement of URR‐driven luciferase activity (HPV16 and 18), indicating the importance of CREB1 in Forskolin/PKA‐stimulated URR activity. Our results also demonstrated that CREB1 can bind AP‐1 sites within the URR to upregulate the transcription of HPV early genes. Taken together, CREB1 appears to be a pivotal driver of HPV early gene transcription.

In conclusion, we propose an HPV/CREB1 positive feedback loop whereby HPV drives CREB1 expression by downregulating miR‐203a and CREB1 activity via the induction of MAPK/MSK signaling, and CREB1, in turn, induces HPV early gene transcription. We therefore demonstrate a novel regulatory network controlled by HPV to regulate proliferation, migration and EMT in cervical cancer (Figure 6H). Going forward, as CREB1 has the potential to regulate the transcription of thousands of genes, the identification of which CREB1‐depedent genes contribute to productive infection and ultimately cervical cancer, will need to be further investigated.

## AUTHOR CONTRIBUTIONS

*Conceptualization*: Yigen Li and Andrew Macdonald; *Formal analysis*: Yigen Li, Ethan L. Morgan, Molly R. Patterson, Emma L. Ryder, Christopher W. Wasson, and Diego Barba‐Moreno; *Investigation*: Yigen Li, Ethan L. Morgan, Molly R. Patterson, Christopher W. Wasson, Emma L. Ryder, and Diego Barba‐Moreno; *Methodology*: Yigen Li, Ethan L. Morgan, Molly R. Patterson, Emma L. Ryder, Christopher W. Wasson, and Diego Barba‐Moreno; *Writing ‐ original draft*: Yigen Li; *Writing – review & editing*: Yigen Li, Ethan L. Morgan, Molly R. Patterson, James A Scarth, Miao Wang, and Andrew Macdonald; *Resources*: Andrew Macdonald; *Supervision*: Andrew Macdonald; *Project administration*: Andrew Macdonald; Funding acquisition, Andrew Macdonald.

## CONFLICT OF INTEREST STATEMENT

The authors declare no conflicts of interest.

## Supporting information


**Figure S1 A)** Western blot analysis of A‐CREB overexpression in indicated cells; **B)** qPCR analysis of CREB1‐dependent genes in HeLa transfected with A‐CREB; **C)** Western blot for PARP cleavage. CREB blot demonstrates successful siRNA knockdown. GAPDH serves as a loading control. **D)** Western blot for PARP cleavage. GAPDH serves as loading control. **E)** Annexin V assay in scramble and CREB siRNA knockdown cell lines. **F)** Cell migration was evaluated by wound healing analysis in indicated cell lines transfected with siCREB1 or A‐CREB. Data shown are mean ± SD.

**Figure S2 A)** Western blot analysis of CREB1 and pCREB1 expression in C33A transfected with GFP‐18E6 or E7; **B)** Western blot analysis of overexpressing GFP‐18E6 or E7, or GFP‐16E6 or E7 in HEK293T. **C)** qPCR analysis of CREB1‐dependent genes in NHK and HPV18‐containing keratinocytes.

**Figure S3 A)** GSE30656 dataset analysis of miR‐203a expression in CC tissue was compared to CN tissue; **B)** GSE19611 dataset analysis of miR‐203a expression was compared among the different abnormalities of cervical cells, low‐grade squamous intraepithelial lesion (LSIL, CIN1), high‐grade squamous intraepithelial lesion (HSIL, CIN2/3) and cervical squamous cell carcinoma (SCC); **C)** qPCR analysis of miR‐203a overexpression in indicated cells; **D)** Western blot analysis of A‐CREB and CREB1 overexpression in HEK293T. Data shown are mean ± SD, n = 3. **, p < 0.01; ***, p < 0.001.

Supporting information.

## Data Availability

The data that support the findings of this study are available from the corresponding author upon reasonable request.
