# Supplementary figures and images for: CREB1 activation promotes human papillomavirus oncogene expression and cervical cancer cell transformation
Source: J Med Virol. 2023 Aug 11;95(8):e29025. doi: 10.1002/jmv.29025 (PMC10952218; doi:10.1002/jmv.29025)

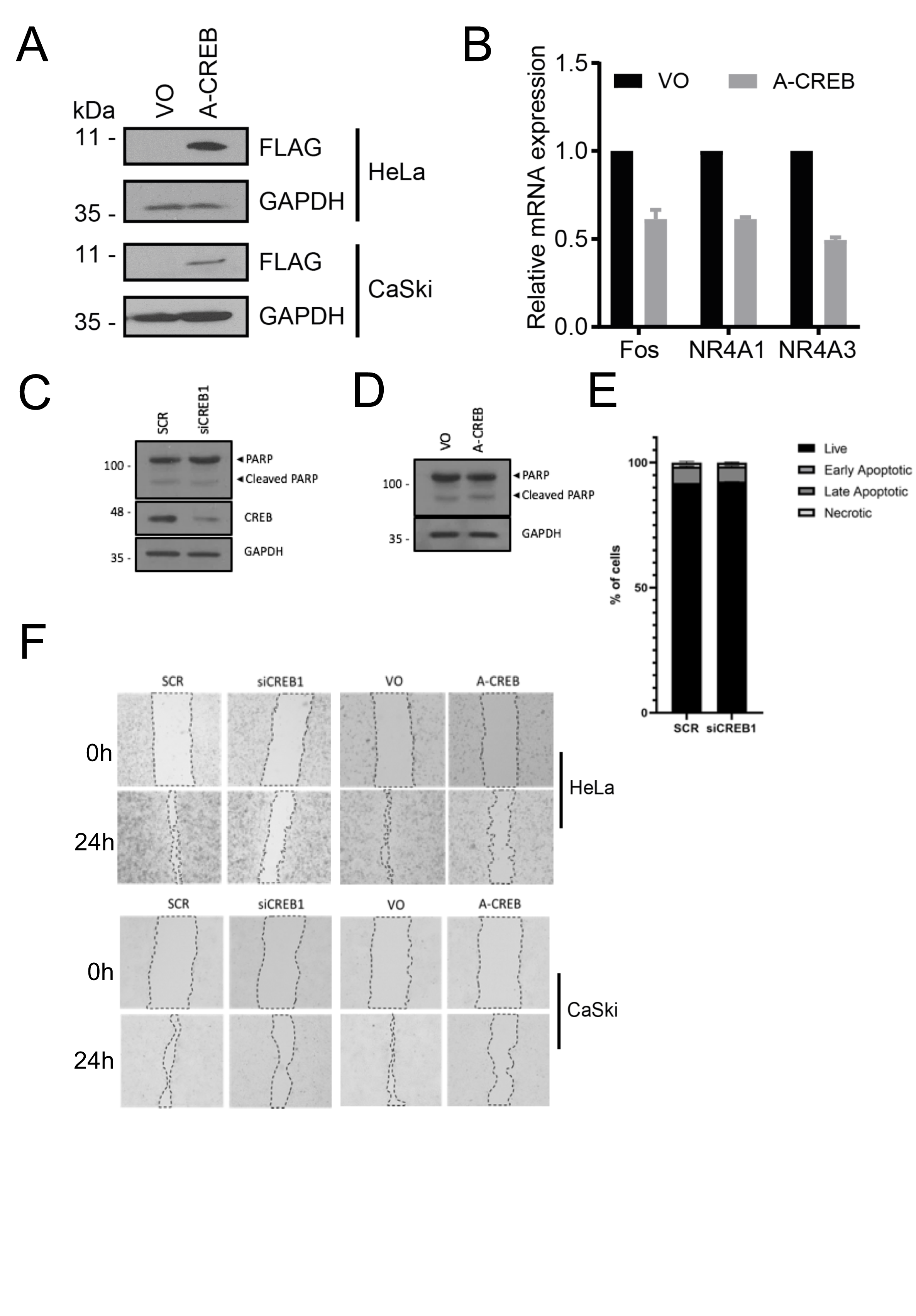

Supplement: Supplementary file 1 — Figure S1 A) Western blot analysis of A‐CREB overexpression in indicated cells; B) qPCR analysis of CREB1‐dependent genes in HeLa transfected with A‐CREB; C) Western blot for PARP cleavage. CREB blot demonstrates successful siRNA knockdown. GAPDH serves as a loading control. D) Western blot for PARP cleavage. GAPDH serves as loading control. E) Annexin V assay in scramble and CREB siRNA knockdown cell lines. F) Cell migration was evaluated by wound healing analysis in indicated cell lines transfected with siCREB1 or A‐CREB. Data shown are mean ± SD. [file JMV-95-0-s004.tif]

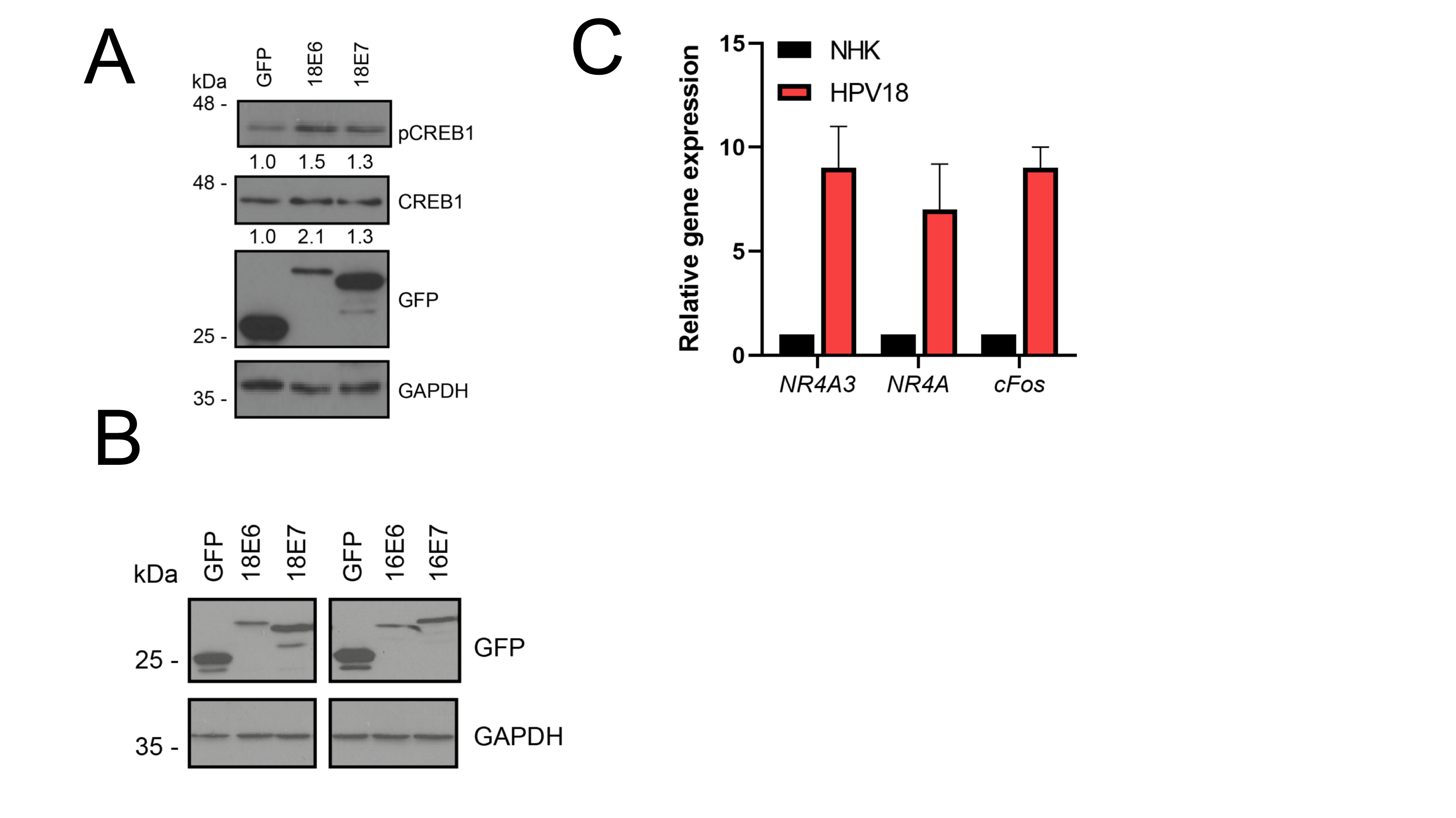

Supplement: Supplementary file 2 — Figure S2 A) Western blot analysis of CREB1 and pCREB1 expression in C33A transfected with GFP‐18E6 or E7; B) Western blot analysis of overexpressing GFP‐18E6 or E7, or GFP‐16E6 or E7 in HEK293T. C) qPCR analysis of CREB1‐dependent genes in NHK and HPV18‐containing keratinocytes. [file JMV-95-0-s002.tif]

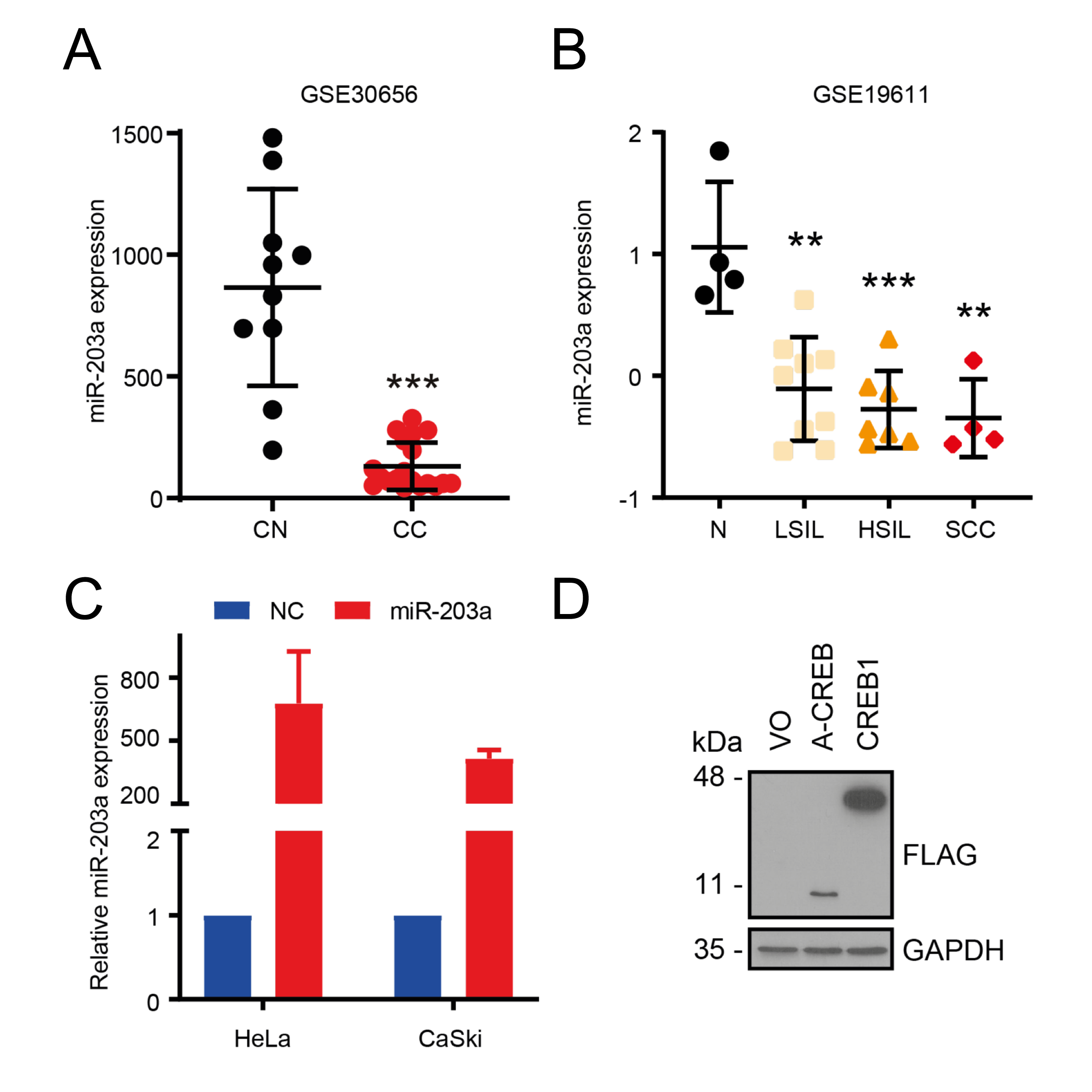

Supplement: Supplementary file 3 — Figure S3 A) GSE30656 dataset analysis of miR‐203a expression in CC tissue was compared to CN tissue; B) GSE19611 dataset analysis of miR‐203a expression was compared among the different abnormalities of cervical cells, low‐grade squamous intraepithelial lesion (LSIL, CIN1), high‐grade squamous intraepithelial lesion (HSIL, CIN2/3) and cervical squamous cell carcinoma (SCC); C) qPCR analysis of miR‐203a overexpression in indicated cells; D) Western blot analysis of A‐CREB and CREB1 overexpression in HEK293T. Data shown are mean ± SD, n = 3. **, p < 0.01; ***, p < 0.001. [file JMV-95-0-s001.tif]

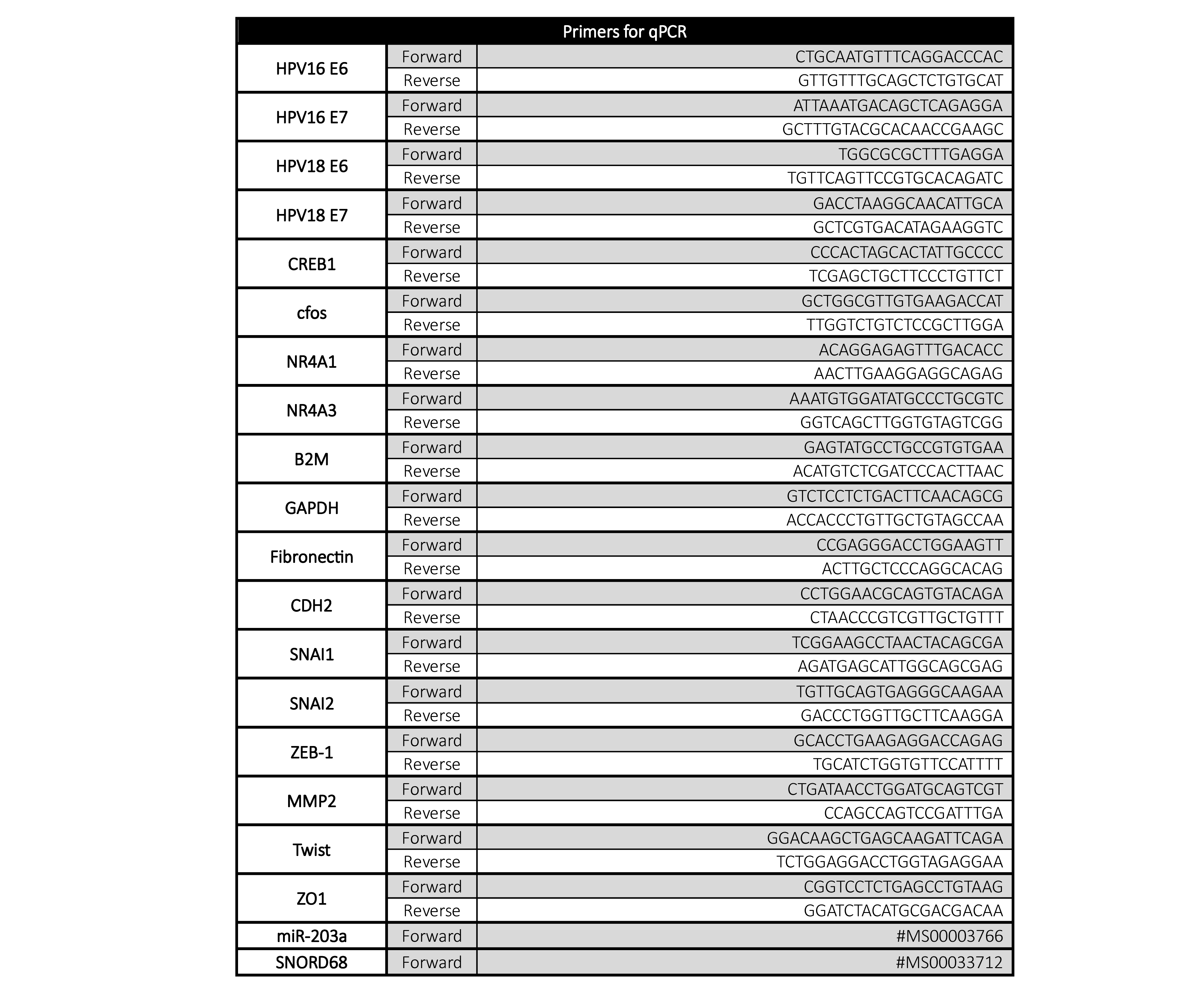


Supplementary Table 1. Table of primer sequences used in this study.

Supplement: Supplementary file 4 — Supporting information. [file JMV-95-0-s003.docx]
